# Uses of generative AI by non-clinician staff at an academic medical center

**DOI:** 10.1038/s44401-025-00063-y

**Published:** 2026-02-02

**Authors:** Kameron C. Black, William J. Haberkorn, Stephen P. Ma, Hanna Kiani, Aditya Bhasin, Jonathan H. Chen, Nigam H. Shah, Keith Morse

**Affiliations:** 1 Department of Medicine, Stanford University School of Medicine, Stanford, CA USA; 2 Department of Anesthesiology, Perioperative, and Pain Medicine, Stanford School of Medicine, Stanford, CA USA; 3 Department of Pediatrics, Stanford University School of Medicine, Stanford, CA USA; 4 Division of Hospital Medicine, Stanford University School of Medicine, Stanford, CA USA; 5 Stanford Division of Computational Medicine, Stanford University, Stanford, CA USA; 6 Clinical Excellence Research Center, Stanford University, Stanford, CA USA

**Keywords:** Business and industry, Health care, Mathematics and computing, Medical research

## Abstract

Large language model (LLM) chat tools have the potential to transform healthcare workflows by improving efficiency and reducing administrative burdens. While prior research has predominantly focused on clinicians, non-clinician healthcare staff constitute the majority of the workforce, and their real-world chat tool use remains uncharacterized. This retrospective, cross-sectional study analyzed de-identified chat logs from a secure, HIPAA-compliant LLM chat tool deployed at an academic medical center over an 11-month period. Among 30,503 chat threads analyzed, 98% originated from non-clinician users across 239 roles. Usage was dominated by administrative tasks including email and document writing (53.9%), text manipulation (9.1%), and brainstorming (6.7%). A notable proportion of interactions included off-label queries unrelated to work or organizational goals, including 5.9% involving clinical decision-making. These findings highlight the need for targeted training, tailored governance policies, and refined evaluation frameworks to optimize appropriate LLM use while mitigating risks in healthcare settings.

## Introduction

Large language models (LLMs) are rapidly being integrated into healthcare^1–6^, with the potential to significantly transform clinical workflows, enhance decision support, and streamline administrative processes^7^. Recent studies highlight the promise of these advanced AI tools to improve efficiency^8^, reduce clinician burden^9^, and potentially enhance patient outcomes through more effective information management and communication support^9–12^. However, the vast majority of existing research has focused primarily on physicians and clinical personnel^13^, when physicians represent only one in 25 employees in U.S. healthcare^14^. There remains limited empirical evidence regarding the specific LLM applications most commonly utilized by non-clinician healthcare staff, such as administrative assistants, case managers, and interpreters^15^.

Without clear insights into how non-clinicians interact with these tools, healthcare organizations face difficulties in optimizing implementations^16^, ensuring patient safety, and proactively managing emerging risks associated with AI-driven technologies^15,17^. To address this critical knowledge gap, our study provides a quantitative analysis of non-clinician usage logs from a secure LLM deployment within an academic medical center. This research aims to equip health system leaders, policymakers, and technology developers with actionable insights to maximize the benefits and mitigate the risks of integrating LLMs into health systems.

## Results

### Usage Trends Across Categories

A total of 30,503 chat threads were analyzed, with 26,691 threads originating from frequent users. Ten primary categories of LLM usage were identified: email and document writing, text manipulation, brainstorming, general information, medical questions, technical support, patient communication, coding, language translation, and image generation. These categories, along with their definitions and representative examples, are detailed in Table 1.

**Table 1:** List of categories with associated definitions and examples

		MedHELM Task Taxonomy		
**Description**	**Example**	**Category**	**Subcategory**	**Task** *if there was a task that was not represented in MedHELM, a new task to be added to the framework was proposed.
Generating code from a description or debugging existing code.	“Can you help me write a Python function that takes a list of numbers and returns the sum of all even numbers in the list?”	Administration & Workflow	Organizing Workflow Processes	*Coding
Providing assistance with software installation, troubleshooting computer issues, and accessing systems or tools.	“I’m having trouble installing the latest version of Adobe Photoshop on my Windows 10 computer. Can you help me figure out what’s going wrong?”	Administration & Workflow	Organizing Workflow Processes	*Technical support and IT issues
Assisting with writing, rewriting, or improving emails, documents, and other forms of written communication.	“Could you help me draft an email to my manager requesting time off next week?”	Administration & Workflow	Organizing Workflow Processes	*Email and document writing
Handling inquiries related to communication with patients and families.	“Can you help me draft a MyChart message to inform a patient’s family about the upcoming surgery and what they should expect during the recovery process?”	Patient Communication & Education	Patient-Provider Messaging	—
Assisting with medical or health information including answering questions about medical conditions, procedures, and pharmaceutical information.	“My patient has been diagnosed with high blood pressure, and there are two different recommended medications. List the side effect profile of each option so I can make an informed decision.”	Clinical Decision Support	Providing Clinical Knowledge Support	Answer medical knowledge questions
Providing answers to general knowledge questions without referencing specific sources.	“What is the capital city of France?”	Administration & Workflow	Organizing Workflow Processes	Handle information requests
Summarizing, interpreting, synthesizing or performing a specific task over a provided text.	“Can you summarize the main points of this article about climate change for me?”	Administration & Workflow	Organizing Workflow Processes	*Text Manipulation
Translating non-clinical text from one language to another.	“Can you translate the following sentence into French: ‘The weather is beautiful today.’ ”	Administration & Workflow	Organizing Workflow Processes	Process referrals/documents
Generating ideas and suggestions on a particular topic or goal or offering guidance on how to handle specific situations.	“[Generate] interviewee phone screening questions for Medical Assistant.”	Administration & Workflow	Care Coordination and Planning	*Brainstorming
Creating images, infographics, and other visual content based on inquiries.	“Can you help me design a logo for my new bakery? I want it to have a vintage feel with pastel colors and maybe some floral elements.”	Patient Communication & Education	Enhancing Accessibility	Generate visual aids
Anything that does not fall under the defined categories	“Create a 16 character password.”			Other

For a complete list of examples given to the LLM, please see the supplementary information.

**Fig. 1:**
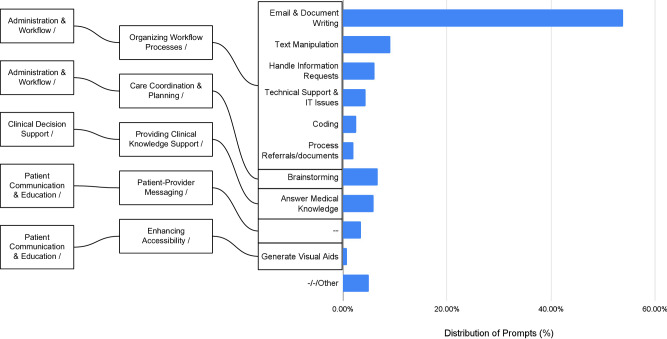
Chat tool usage by task category, mapped to the MedHELM task taxonomy. Horizont albars indicate the percentage of conversation threads assigned to each task category (x-axis, distribution of prompts [%]). Boxes on the left depict the corresponding MedHELM category and subcategory for each task, with connecting lines indicating the mapping.

Analysis of usage patterns demonstrated that ‘Email & Document Writing’ tasks dominated, representing nearly 53.9% of total activity. Other prominent categories included ‘Text Manipulation’ (9.1%), ‘Brainstorming’ (6.7%), ‘Handle Information Requests’ (6.1%), and ‘Answer Medical Knowledge Questions’ (5.9%). ‘Technical Support & IT Issues’ (4.4%), ‘Patient-Provider Messaging’ (3.5%), ‘Coding’ (2.6%), ‘Process Referrals/ Documents’ (2.0%), and ‘Generate Visual Aids’ (0.8%) comprised the remainder of usage (Fig. 1).

### Summary of User Role Categories in the Study Sample

Of the users who signed in to their department and had a role available to our system (comprising 97.2% of total users), 98.0% were non-clinicians. In this study, the term ‘department’ referred to distinct clinical entities, typically different ambulatory clinic locations or clinical services, that require unique builds within the electronic health record. Please see the Supplementary Information (Supplementary Fig. 2) for details regarding the distribution of user role categories, which were manually mapped to high-level U.S. Standard Occupational Classification (SOC) Major Groups^18^. The complete taxonomy mapping of usage categories to the MedHELM framework is detailed in Supplementary Table 2 (see Supplementary Information C for advanced practice provider and allied health professional definitions).

### Illustrative Examples from Quantitative Usage Categories

In addition to quantifying aggregate usage patterns, examples of user queries were reviewed that depict the potential value and risks of secureLLM deployment. An example of this being requests to automate frequently repeated administrative tasks, such as:

*“user: please help me write a brief summary of what a spider angioma is for patients in my pediatric dermatology clinic”*

*“user: ask me questions to better generate a script for our schedulers to inform families of our wait times before scheduling.”*

However, our analysis also surfaced conversations largely unrelated to work or organizational goals. These included:

*“user: guess that baby shower game tagline”*

*“user: can i know the price of the stock tesla 2025?”*

*“user: what is a good joke for today?”*

Despite the majority of user roles being non-clinical, a significant proportion of prompts were related to clinical decision making. Examples included:

*“user: What are the symptoms for RSV?”*

*“user: Patient taking high dose oxcarbazepine, valproic acid medium dose valproic acid and cenobamate. Presents with constipation and abdominal pain as well as decreased appetite. What is the differential diagnosis?”*

*“user: Write a letter of medical necessity for patient to get Monogenic Hypertension Evaluation testing through Athena Diagnostics*.

*“user: write letter for appeal to CVS Caremark to approve Retacrit for patient.”*

Another use case was related to the generation of insurance prior authorization requests, detailing medical necessity of certain treatments.

## Discussion

This study contributes an early quantitative analysis of real-world chat tool use among non‑clinician healthcare staff, addressing a gap in the existing literature that has focused almost exclusively on clinicians. Our analysis of 30,503 chat threads moves beyond prior survey-based or pre-categorized approaches^5,6^, revealing how non-clinicians adopt and integrate these LLM tools.

Usage was dominated by tasks, such as email composition and document preparation, suggesting considerable untapped potential for more sophisticated applications. Expanding staff awareness of the range of workflow-relevant functions, beyond traditional search or basic writing tasks, alongside training in prompt engineering^16^ and the development of customized templates for high‑frequency administrative tasks, such as prior authorization letters^19^, could improve efficiency and reduce administrative burden. However, the presence of prompts requiring nuanced clinical judgment underscores the need for role‑appropriate education and governance^20^ to prevent out‑of‑scope use.

Unexpected usage patterns included a notable proportion of non-work related personal queries (i.e., trivia queries, creative writing), which at scale may generate unnecessary computational and environmental costs^21^. In addition, prompts requiring nuanced clinical judgment were observed. This likely represents the small proportion of clinicians engaged with the system despite role-targeted alternatives, or non-clinicians involved in workflows related to insurance. Yet governance and risk-management considerations necessitate further defining non-clinician scope with regard to seeking LLM-derived medical guidance. Several non-clinical uses were identified, including Email and Document Writing, Coding, Technical Support and IT Issues, Text Manipulation, and Brainstorming, that were absent from the MedHELM task taxonomy. This gap illustrates limits in existing frameworks’ ability to capture real‑world LLM uses among non‑clinician staff. We propose the addition of these tasks to MedHELM version 2 under the ‘Administration & Workflow’ category.

These findings highlight the importance of deployment strategies and monitoring tailored to departmental needs. Training priorities will differ, for example, between language translation teams focused on the LLM’s language strengths and information technology (IT) groups leveraging coding assistance features or internal IT workflow troubleshooting capabilities. Role‑based analyses are essential for determining appropriate user education that includes insights into job-specific workflow enhancements, such as prompting techniques for document insight generation or best practices regarding programming assistance.

Limitations include the single‑center design, the 11‑month study period, and potential classification challenges due to multi‑intent conversations. Inconsistent role designations also limited role‑specific analyses. Future research should link usage patterns to departmental context, refine occupational coding, and explore automating high‑volume administrative tasks, such as prior authorization or patient education document creation.

Based on these findings, we offer three targeted policy recommendations for future LLM deployments in healthcare, each linked to specific patterns that were observed:

1. *Implement real-time dashboards for usage monitoring*. Both directly valuable workflow applications (i.e., automating documentation) and unrelated or inappropriate use (e.g., personal queries, off‑task interactions) were observed. Continuous visibility into usage patterns would allow health systems to quickly identify emerging high‑impact applications worth expanding and to intervene early when unrelated, risky, or non‑compliant prompts are used. We recommend institutions deploy analytics tools within the secure LLM environment to display usage by category, department, and frequency, with automated alerts for unusual trends (i.e., spikes in clinical decision support queries from non‑clinical departments).

1. *Develop role‑ and department‑specific, guidance and educational resources*. Despite 98% of users being non‑clinicians, a notable subset of prompts involving nuanced clinical decision‑making or insurer‑facing medical necessity statements. Tailoring guidance to user roles would help prevent out‑of‑scope activity, reduce risk, and control compute costs. We recommend organizations map common task categories by department and deliver targeted education that clarifies appropriate use of secure LLMs.
2. *Automate validated use cases*. Many users repeated specific tasks (such as patient education or insurance-related communications). Automating high-value use cases can enable further efficiency gains. We recommend institutions validate specific workflows by creating automations and templates that encourage use and simultaneously educate users on a broader range of chat tool capabilities.

By systematically monitoring real-world usage patterns and proactively managing risks, health systems can maximize the benefits of LLM integration while safeguarding patient safety and operational efficiency.

## Methods

### Study Design and Setting

This retrospective, cross-sectional study was conducted at Stanford Medicine Children’s Hospital, leveraging de-identified usage logs of user prompts from a secure, HIPAA-compliant LLM chat tool (GPT-4o, OpenAI). The study period spanned from April 22, 2024 to February 28, 2025 and encompassed a total of 30,503 conversation threads across 239 clinical and administrative roles. For the purposes of this study, the clinician role was defined as one that provides direct patient care in the form of diagnosis, treatment and prescribing (including physicians, APPs, certified nurse midwife, certified nurse practitioner, clinical nurse specialist, and certified registered nurse anesthetist)^22^. Roles of non-clinicians spanned functions in management, training, nursing, education, administrative support, IT services, and more. For a more detailed breakdown of non-clinician user roles by category, see Supplementary Fig. 2 in Supplementary Information. Of note, most physicians are employed by the affiliated university and are granted access to and encouraged to use a different secure LLM chatbot.

This study was reviewed and deemed exempt by the Stanford University Institutional Review Board (Protocol ID: 81541).

### Sample Selection

To focus on users with greater familiarity and engagement with the platform, our primary analysis was restricted to threads generated by “frequent users,” defined as individuals with more than five recorded interactions with the chat tool. This threshold was chosen to ensure an adequate sample size, as very few users had surpassed five interactions early in deployment (20.7% in September 2024) and this proportion increased to 43.7% by the end of the study period. Study size was determined by the number of chat threads available at the time of the data analysis.

### Categorization Scheme Development

The categorization scheme for user queries was developed through an iterative process, initially adapting categories from Bedi et al.^15^ to reflect healthcare-specific workflows. The preliminary category list was comprised of 11 categories, including an “other” designation for uncaptured use cases. Each human reviewer independently applied these labels to a random sample of 100 messages, followed by consensus labeling sessions to resolve discrepancies and refine categories and their associated definitions.

To generate example messages for each category, a secondary LLM was prompted to produce five candidate messages per category. These were then classified by this LLM to ensure alignment, and independently reviewed by two project team members (WH and KB), who selected the three most representative examples for each category. When a conversation was labeled as “other,” the secondary LLM suggested a descriptive category name, which informed subsequent category list iterations. A curated set of examples was subsequently used to guide both manual and automated classification of conversation threads. The final category list, including definitions and representative examples, is detailed in Table 1 and the Supplementary Information.

### Classification Process

Classification of user queries employed a hybrid human-AI approach. Three independent physician reviewers (WH, KB, SM) manually labeled random samples of conversation threads. Discrepancies were resolved by consensus or, when necessary, adjudication by a third reviewer. Automated classification was performed using the GPT-4o API. Model prompts and category definitions were iteratively refined based on error analysis and reviewer feedback (please see Supplementary Information A for detailed prompt methodology and categorization examples in Supplementary Table 1). After this process was completed, author KB manually mapped category results to the MedHELM task taxonomy^23^.

### Validation and Reliability Assessment

Detailed validation methodologies and performance metrics are provided in Supplementary Information B, with categorization performance visualized in Supplementary Fig. 1.

### Data Handling and Preprocessing

All conversations were preserved in their entirety as single input sequences to the LLM, and included only the user prompts. Threads exceeding 32,000 characters, the maximum row limit for Excel, were truncated which affected a small proportion (1.1%) of the dataset.

## Supplementary information

npj_Black_Supplementary Information (1)

## Data Availability

The datasets generated and/or analyzed during the current study are not publicly available due to the data being obtained from internal systems and containing patient, personal, and/or proprietary information that is not in their current form appropriate for public posting, but are available from the corresponding author on reasonable request.

## Code availability

Classification was performed using the GPT-4o API with a temperature setting of zero. All model responses were returned in JSON format for reliability, with any errors adjudicated by a secondary model. Please see Supplementary Information A for the categorization prompt used, along with example instructions for formatting responses in JSON.
