# Supplementary material for: Uses of generative AI by non-clinician staff at an academic medical center
Source: Npj Health Syst. 2026 Feb 2;3:13. doi: 10.1038/s44401-025-00063-y (PMC13354237; doi:10.1038/s44401-025-00063-y)
Supplement: Supplementary file 1 — npj_Black_Supplementary Information (1) [file 44401_2025_63_MOESM1_ESM.pdf]

Supplementary Information A. Categorization prompt

Here is the specific prompt used:
You are specialized in categorizing user intent in chatbot conversations that could be used to understand user utilization. You will be provided with categories and user messages from a conversation, and your goal is to categorize the conversation as a whole. Please only consider the user's underlying intent and what they needed help with. If the conversation does not clearly fall under one of the provided categories, write 'Other'. Do not provide an explanation or any other text besides the category name(s). You may provide 1-3 categories. Use the minimum number of categories to capture the conversation.

Format Instructions:

Write your response in JSON format.

```
{
    "categories": List[str] // appropriate category names
}
```

Categories:
{categories}

Supplementary Information B. Validation of Categorization Methodology

The interrater reliability among human reviewers, as measured by Cohen's kappa, ranged from 0.58 to 0.74. Specifically, kappa values were 0.62 (WH & KB), 0.74 (WH & SM), and 0.58 (KB & SM). Automated classification using the LLM relative to reviewer WH achieved an overall accuracy of 0.82, with an area under the precision-recall curve (AUPRC) of 0.59 and an area under the ROC curve (AUC) of 0.85. The model exhibited the highest performance in categorizing Language Translation, Patient Communication, and Technical Support/IT Issues, with particularly strong recall and precision in these categories. The moderate interrater reliability (Cohen's kappa ranging from 0.58 to 0.74) indicates some ambiguity in category definitions and overlap between use cases, reflecting the inherent complexity in classifying real-world LLM interactions.

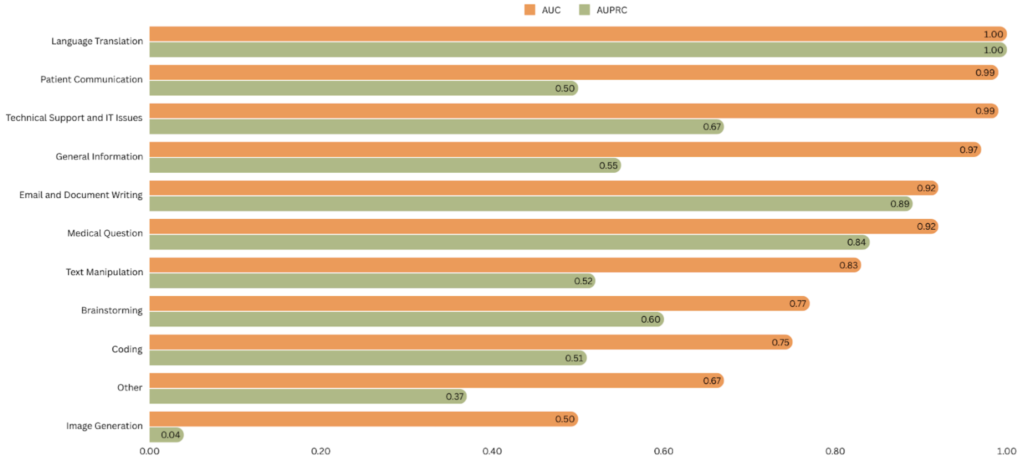

Supplementary Figure 1. AskDigi categorization performance by AUC, AUPRC.

Category	Description	Examples
Coding	Generating code from a description or debugging existing code.	"Write a function to calculate the factorial of a number in Haskell.", "Write a function in Ruby to check if a string is a palindrome.", "How do I implement the Fibonacci sequence using recursion in C++?"
Technical Support and IT Issues	Providing assistance with software installation, troubleshooting computer issues, and accessing systems or tools.	"My external hard drive is not recognized by my computer.", "I can't access shared folders on the network.", "My webcam isn't detected. How do I fix this?"
Email and Document Writing	Assisting with writing, rewriting, or improving emails, documents, and other forms of written communication.	"Draft a letter requesting financial aid reconsideration from the university.", "Write an apology letter to a friend I had an argument with.", "Write a letter requesting a refund from a service provider."
Patient Communication	Handling inquiries related to communication with patients and families.	"How should I approach a difficult conversation with a patient's family about prognosis?", "How can I ensure informed consent when a patient

		seems confused about a procedure?", "How do I communicate a new diagnosis to a patient who may not understand medical jargon?"
Medical Question	Assisting with medical or health information including answering questions about medical conditions, procedures, and pharmaceutical information.	"What are the signs of vitamin D deficiency?", "What are the signs of early menopause?", "What is the recommended dosage for vitamin D?"
General Information	Providing answers to general knowledge questions without referencing specific sources.	"Who was Julius Caesar?", "Who were the Mayans?", "Who were the Vikings?"
Text Manipulation	Summarizing, interpreting, synthesizing or performing a specific task over a provided text.	"Extract all the names and places mentioned in this passage.", "Identify any contradictions in this passage.", "Summarize the key arguments in this debate transcript."
Language Translation	Translating text from one language to another.	"Provide the Arabic translation of 'When is the next bus arriving?'", "Provide the German translation for 'Where can I find a taxi?'", "Translate 'He who laughs last laughs best' into German."
Brainstorming	Generating ideas and suggestions on a particular topic or goal or offering guidance on how to handle specific situations.	"What are some tips for starting a successful podcast?", "What are some novel approaches to teaching history to high school students?", "What are some strategies to save money while traveling?"
Image Generation	Creating images, infographics, and other visual content based on inquiries.	"Produce an isometric illustration of a city block.", "Generate an animated GIF of a spinning globe.", "I need

		an avatar for my gaming profile.”

Supplementary Table 1. List of original categories with associated descriptions and additional examples used as context for chat tool categorization process.

	**MedHELM Task Taxonomy**		
**Category**	**MedHELM Category**	**MedHELM Subcategory**	**MedHELM Task** (*if there was a task that was not represented in MedHELM, we proposed a new task to be added to the framework.)
Coding	Administration & Workflow	Organizing Workflow Processes	*Coding
Technical Support and IT Issues	Administration & Workflow	Organizing Workflow Processes	*Technical support and IT issues
Email and Document Writing	Administration & Workflow	Organizing Workflow Processes	*Email and document writing
Patient Communication	Patient Communication & Education	Patient-Provider Messaging	— (formerly ‘Patient communication’)
Medical Question	Clinical Decision Support	Providing Clinical Knowledge Support	Answer medical knowledge questions (formerly ‘Medical question’)
General Information	Administration & Workflow	Organizing Workflow Processes	Handle information requests (formerly ‘General information’)
Text Manipulation	Administration & Workflow	Organizing Workflow Processes	*Text Manipulation
Language Translation	Administration & Workflow	Organizing Workflow Processes	Process referrals/documents (formerly ‘Language translation’)

Brainstorming	Administration & Workflow	Care Coordination and Planning	*Brainstorming
Image Generation	Patient Communication & Education	Enhancing Accessibility	Generate visual aids (formerly ‘Image Generation’)
Other	–	–	Other

Supplementary Table 2. Category results manually mapped to the MedHELM task taxonomy.

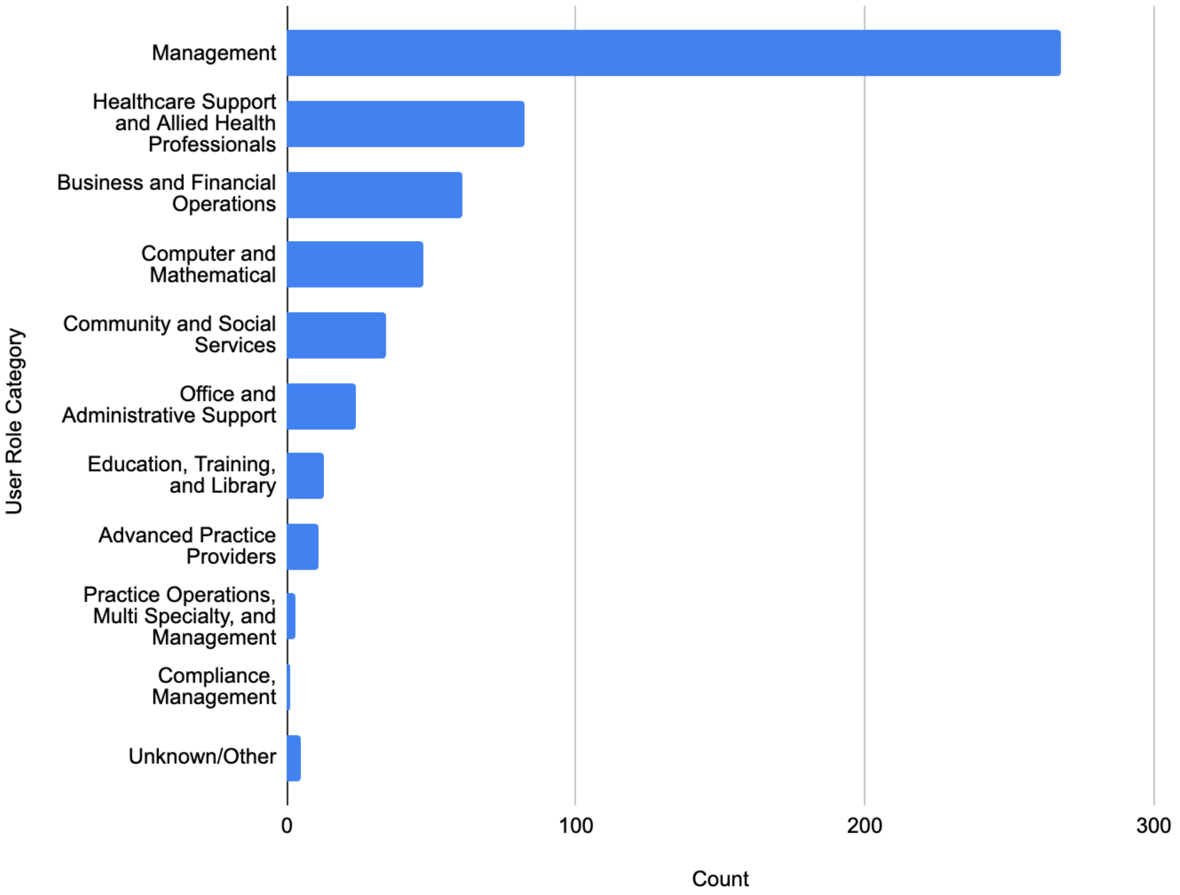

Supplementary Figure 2. Distribution of Non-Clinician User Roles by Occupational Category. User roles were manually mapped to one of the high-level U.S. Standard Occupational Classification (SOC) Major Groups[1] and further refined to incorporate the AUA Consensus Statement[2] definitions of "Advanced Practice Providers" and "Allied Health Professionals".

Supplementary Information C. Advanced Practice Provider and Allied Health Professional Definitions

In this study, we adhered to the AUA Consensus Statement on Advanced Practice Providers[2]: "As of February 2014 the AUA recognizes advanced practice registered nurses (APRN) and PAs as 'advanced practice providers (APPs)'. The term 'allied health professional' includes nurses, medical technicians, and assistants…'APRN' is a term that covers four distinct roles: certified nurse practitioner, certified nurse midwife, clinical nurse specialist, and certified registered nurse anesthetist."

References

1. U.S. Bureau of Labor Statistics. *Standard Occupational Classification Manual (2018 Edition)*. https://www.bls.gov/soc/2018/soc_2018_manual.pdf (2018).
2. AUA Ad Hoc Work Group on Advanced Practice Providers Chris M. Gonzalez MD, MBA, Timothy Brand MD, Lou Koncz PA-C, Ken Mitchell MPAS, PA-C, Aaron Spitz MD, Susanne Quallich ANP-BC, NP-C, CUNP, FAANP, Tim Irizarry MS, NREMT-P, PA-C, Jonathan Rubenstein MD, Pablo J. Santamaria MD, FACS, Howard Snyder MD, FACS, John Gore MD, Christopher Porter MD, Kathleen M. Zwarick PhD, CAE and John Kristan. AUA Consensus Statement on Advanced Practice Providers: Executive Summary. *Urol. Pract.* **2**, 219–222 (2015).
